# Editorial: Advances in understanding the nature and features of misophonia

**DOI:** 10.3389/fnins.2023.1267682

**Published:** 2023-09-12

**Authors:** M. Zachary Rosenthal, Julia Campbell, Cara Altimus

**Affiliations:** ^1^Duke University Medical Center, Duke University, Durham, NC, United States; ^2^Department of Speech, Language, and Hearing Sciences, University of Texas-Austin, Austin, TX, United States; ^3^Milken Institute Center for Strategic Philanthropy, Washington, DC, United States

**Keywords:** misophonia definition, misophonia measurement, misophonia phenotype, misophonia treatment, misophonia neuroscience

Welcome to the first Research Topic in any peer-reviewed journal dedicated to the topic of misophonia. What is misophonia? That is a—if not *the*—primary question driving this collection of work. Misophonia is a newly studied clinical presentation characterized by intolerance of certain aversive sounds and associated cues. These are typically repetitive oral (e.g., chewing, swallowing) or nasal (e.g., sniffling, heavy breathing) stimuli made by other people, but can include environmental sounds and those made by animals. When encountering these sounds, often called “triggers,” a range of bottom-up (i.e., automatic) and top-down (i.e., cognitively mediated) responses have been observed, including those across neural (e.g., enhanced connectivity between orofacial and auditory cortical regions; Kumar et al., 2021), autonomic (e.g., heightened heart rate and skin conductance in response to trigger sounds; Kumar et al., 2017), and perceptual (e.g., Samermit et al.) systems. According to Swedo et al., responses can elicit significant psychological distress and impairment in functioning and cannot be better accounted for by other disorders that feature reactivity to certain auditory cues (e.g., other decreased sound tolerance disorders diagnosed by audiologists such as tinnitus and hyperacusis, sensory processing and modulation disorders commonly diagnosed by occupational therapists, or psychiatric disorders diagnosed by mental health providers such as autism spectrum or anxiety disorders).

Jastreboff and Jastreboff (2001) coined the term misophonia, and the first small yet influential research studies were published 12 years later (Edelstein et al., 2013; Schröder et al., 2013). From 2013 until 2019, before the launch of the Misophonia Research Fund (https://misophoniaresearchfund.org), research studies were published at a slow rate and, though pioneering and influential, often suffered from significant methodological weaknesses that precluded clear inferences or conclusions. Since 2020, the pace and quality of scientific research has significantly increased. Today, researchers from around the world across scientific and clinical disciplines are actively working to discover insights about the nature and features of misophonia.

What follows are 23 articles, 18 of which are empirical studies that break ground in the measurement, biological underpinnings, and phenotypic characterization of misophonia. If one were to read the whole Research Topic, we might advise they begin with Swedo et al. Why? Swedo et al. outline the method and results from a structured expert consensus process that resulted in the first definition of misophonia offered outside of a single research group or clinician. This major advance for the field promises over time to become a seminal publication with lasting impact. A second article to read could be Brout, who thoughtfully offers a commentary about the methodology used in Swedo et al.. Brout highlights the need for ongoing attention to modifications and iterations in the definition of misophonia, suggesting this process be ongoing and include multi-disciplinary research and a more diverse expert panel.

## Conceptual models

Next, it could be helpful to read the three conceptual papers, to gain a sense of history and perspective about misophonia. Jastreboff and Jastreboff, original pioneers in the field, summarize a neurophysiological model emphasizing the role of several basic human processes (e.g., learning, memory, and emotion) in the possible etiology and maintenance of misophonia. In addition, they describe a history of clinical observations using Tinnitus Retraining Therapy (TRT; Jastreboff and Jastreboff, 2014) as a treatment approach. No randomized controlled trials have been conducted examining this treatment for misophonia, but the authors describe having an extensive amount of uncontrolled clinical results. Indeed, for TRT to be considered an evidence-based treatment for misophonia, it will be essential that researchers evaluate the efficacy of this treatment in a controlled manner. In doing so, the proposed treatment mechanisms can be tested to evaluate the core tenets of the neurophysiological model.

Neacsiu et al. provide a detailed model with testable predictions that can be used to advance clarity about the underlying neural processes in misophonia. In addition, Neacsiu et al. outline a rationale for the use of neurostimulation as a novel intervention, appropriately proposing candidate targets be studied first and validated before interventions are implemented.

Mednicoff et al. review the literature at the intersection of misophonia and musicality. They hypothesize that heightened sensitivities to sounds in the context of music could represent a developmental vulnerability in the etiology of misophonia. This speculation highlights the possibility that a generalized sensitivity to sounds could be one factor in a causal pathway leading to the onset of misophonia in children. After reading these conceptual papers, the reader could then explore the remaining empirical articles in any order of interest.

## Assessment of misophonia

Several papers focused on the assessment of misophonia. In Rinaldi et al., the Sussex Misophonia Scale for Adolescents is introduced as the first self-report measure of misophonia for adolescents with psychometric validation procedures to be published in a peer-reviewed journal. Two other papers conducted cross validation of an established measure of misophonia symptoms (i.e., the S-Five) in Mandarin (Vitoratou et al.) and German (Remmert et al.) samples. With these papers and others, the S-Five is now the most well-studied self-report measure of misophonia.

Williams et al. developed the first self-report measure of misophonia that intentionally aligns with the consensus definition (Swedo et al.). This measure, the Duke-Vanderbilt Misophonia Screening Questionnaire, is capable of briefly screening people to determine clinical caseness. Additional research now is needed to cross-validate the measure and determine its sensitivity and specificity to misophonia.

Assessment of misophonia may best be done using a multi-disciplinary strategy (e.g., mental health, occupational therapy, audiology). In the context of audiologic assessment, Aazh et al. conducted one of the first studies to investigate audiologic factors associated with misophonia. The authors concluded that, when assessing individuals with tinnitus and hyperacusis in audiologic clinical settings, it is important to screen for misophonia. This work will help audiologists gain clarity on the appropriate testing batteries and clinical care pathways to use for patients with misophonia.

## Biological features of misophonia

Several papers in the Research Topic are dedicated to discoveries about biological features of misophonia. Smit et al. reported findings from the first study exploring the genetics of misophonia using a large database from the 23andMe commercial dataset. They reported that a genetic association with a single item assessing rage responses to people eating was associated with tinnitus and certain mental health problems (e.g., generalized anxiety disorder), inversely related to others (i.e., autism spectrum disorder), and unrelated with other disorders (e.g., obsessive-compulsive disorder, attention deficit disorder). No definitive conclusions can be made about the genetics of misophonia from this study; however, this foundational study aligns with the hypothesis that misophonia is related to disorders associated with heightened anxiety.

Two studies investigated neural underpinnings of misophonia. Grossini et al. examined neural systems, finding a central pathway (i.e., auditory-insula-limbic) may be elicited when triggered, initiating downstream sympathetic nervous system activation. These findings are congruent with those of Edelstein et al. (2013) and provide indirect support for Jastreboff and Jastreboff's neurophysiological model.

Hansen et al. provide the first evidence using neuroimaging that non-orofacial triggers may be processed atypically in the brain. These data expand the exciting findings from Kumar et al. (2021) about the possible motor basis of misophonia, while highlighting the important need to conceptualize and study misophonia as a phenomenon that may not be restricted to oral and facial triggers.

Efraim Kaufman et al. explored the underlying biology of misophonia by comparing those with and without misophonia on measures of physical pain and sensory processing. In line with recent results from other investigative teams (Andermane et al., 2022), results from this study indicate misophonia may be linked to a generally increased sensory responsiveness. Importantly, findings from Efraim Kaufman et al. have implications for the formulation of diagnostic criteria, suggesting that misophonia can be differentiated from sensory over-responsivity using behavioral responses to painful and non-painful stimuli.

## Phenotypic features of misophonia

Several papers in the Research Topic aimed to better understand the phenotypic features of misophonia. It is unknown if misophonia is best conceptualized in a homogenously phenotypic manner or, alternatively, whether it is characterized by heterogeneous clinical features across people. Norris et al. examined this question using cluster-based modeling. Findings suggest that some people may have only misophonia whereas others will have these symptoms along with co-occurring diagnoses. This finding is consistent with the suggestion that multi-disciplinary treatments for misophonia may need to be used to account for the presence of co-occurring audiologic and mental health disorders (Brout et al., 2018).

Rosenthal et al. conducted the first comprehensive assessment of medical health history and DSM-5 psychiatric disorders using structured psychiatric diagnostic interviews. Consistent with a recent study with children (Guzick et al., 2023), Rosenthal et al. found that anxiety disorders were the most common current co-occurring mental health problems with misophonia. No clear medical health problems emerged as significantly associated with misophonia severity. These results highlight the importance of treating the co-occurring clinical presentation of misophonia and mental health problems such as anxiety disorders.

Three studies aimed to elucidate underlying processes related to emotional functioning and learning. Dibb and Golding conducted a longitudinal assessment in adults, finding that anger and disgust are more strongly associated with the experience of misophonia than anxiety. They also reported that quality of life in people with misophonia was lower than a general community sample and was similar to individuals with tinnitus. Avoidance of triggers, extent of the emotional reactions, and depression were associated with perceptions of lower quality of life over time in participants with misophonia.

Wang et al. explored which features of misophonia were associated with impairment in functioning. Perceived emotional threat was predictive of worse functional impairment, and this was explained, in part, by negative beliefs about emotions and depression symptoms. Results from Wang et al. provide support for the hypothesis that processes related to emotional functioning are germane to misophonia, and, accordingly, it may be important for treatment studies to identify candidate targets for change attributable to emotional processes (e.g., emotion regulation).

Several studies investigated the role of context, perception, and learning in misophonia. Savard et al. used a masking approach to systematically vary the identification of triggering sounds. The degree to which participants high in misophonia symptoms identified masked sounds as triggers influenced reactivity to these sounds. Similarly, Heller and Smith found that the pleasantness of trigger sounds may be altered when such sounds are associated with certain verbal causal properties or misheard as being in a more pleasant emotional category. Siepsiak et al. used a different approach, finding that responses to auditory triggers could be influenced when presented within a credible visual context for creating such sounds.

Samermit et al. created an open access database (https://osf.io/3ysfh/) with paired videos of sound triggers occurring in either visually congruent (i.e., seeing a person sniffing while hearing a sniffing sound) or plausible but incongruent visual contexts (e.g., seeing a broom pushing dirt while hearing the same sniffing sound). Like Siepsiak et al., the results of this study indicated that reactivity to triggering sounds may be lower when there is an alternative and believable causal source presented within the visual context. These studies all suggest that how one perceives cues and their context could change emotional reactivity to misophonic triggers. The treatment implications are clear: interventions targeting appraisals and cognitive processes may be helpful.

Finally, Ward et al. conducted the first study to begin exploring possible mechanisms of learning associated with misophonia. Using a translational approach, results pointed to the possibility that heightened sensitivity and discrimination learning, but not overgeneralization, may be basic learning processes underlying misophonia severity. This preliminary study paves the way for future scientific advances targeting specific learning processes that account for the onset and maintenance of symptoms in misophonia.

## Summary

Taken together, the articles in this Research Topic reflect a leap forward in understanding the nature and features of misophonia. In this body of work, there are many “firsts” to point out. Examples include: The first expert consensus definition (Swedo et al.), genetics study (Smit et al.), measure for adolescents (Rinaldi et al.), assessment measures in Mandarin (Vitoratou et al.) and German (Remmert et al.), assessment measure aligned with the consensus definition (Williams et al.), comprehensive diagnostic assessment of DSM-5 diagnoses using structured interviews (Rosenthal et al.), and studies discovering that misophonic reactions may be a function of trigger identification (Savard et al.), pleasantness (Heller and Smith), and congruence between trigger source and observable visual contextual information (Samermit et al.; Siepsiak et al.).

The Research Topic comes at a time when misophonia is rapidly gaining attention scientifically. Over the last 2 years more scientific studies investigating misophonia have been published than in all previous years combined. It is hoped that the studies in this Research Topic will help galvanize the field, inspiring more rigorous research across a range of disciplines, methodologies, and perspectives. Ultimately, treatments are needed that have targeted mechanisms of change discovered scientifically, are carefully evaluated in treatment research, and can be readily disseminated to clinicians worldwide in an effort to reduce suffering and enhance the lives of people with misophonia and their loved ones.

## Author contributions

MR: Writing—original draft. JC: Writing—review and editing. CA: Writing—review and editing.
